# Assessing Mobile Phone Digital Literacy and Engagement in User-Centered Design in a Diverse, Safety-Net Population: Mixed Methods Study

**DOI:** 10.2196/14250

**Published:** 2019-08-29

**Authors:** Sarah S Nouri, Patricia Avila-Garcia, Anupama Gunshekar Cemballi, Urmimala Sarkar, Adrian Aguilera, Courtney Rees Lyles

**Affiliations:** 1 Division of General Internal Medicine Department of Medicine University of California, San Francisco San Francisco, CA United States; 2 Department of Psychiatry Zuckerberg San Francisco General Hospital University of California, San Francisco San Francisco, CA United States; 3 Center for Vulnerable Populations Zuckerberg San Francisco General Hospital University of California, San Francisco San Francisco, CA United States; 4 School of Social Welfare University of California, Berkeley Berkeley, CA United States

**Keywords:** health information technology, mHealth, user-centered design, health literacy, digital literacy, limited English proficiency

## Abstract

**Background:**

Health care systems are rapidly deploying digital tools for disease management; however, few studies have evaluated their usability by vulnerable populations. To understand the barriers to app usage among vulnerable populations, we employed user-centered design (UCD) methods in the development of a new text messaging app.

**Objective:**

The study aimed to describe variations in patients’ engagement in the app design process, focusing on limited health literacy (LHL), limited English proficiency (LEP), and limited digital literacy (LDL).

**Methods:**

We conducted 20 in-depth semistructured interviews with primary care patients at a public health care system, used open-ended discussions and card sorting tasks to seek input about mobile phones and text messaging, and used open coding to categorize the patterns of mobile phone usage and to evaluate engagement in the card sorting process. We examined qualitative differences in engagement by examining the extensiveness of participant feedback on existing and novel text messaging content and calculated the proportion of patients providing extensive feedback on existing and novel content, overall and by health literacy, English proficiency, and digital literacy.

**Results:**

The average age of the 20 participants was 59 (SD 8) years; 13 (65%) were female, 18 (90%) were nonwhite, 16 (80%) had LHL, and 13 (65%) had LEP. All had depression, and 14 (70%) had diabetes. Most participants had smartphones (18/20, 90%) and regularly used text messaging (15/20, 75%), but 14 (70%) of them reported having difficulty texting because of inability to type, physical disability, and low literacy. We identified 10 participants as specifically having LDL; 7 of these participants had LEP, and all 10 had LHL. Half of the participants required a modification of the card sorting activity owing to not understanding it or not being able to read the cards in the allotted time. The proportion of participants who gave extensive feedback on existing content was lower in participants with limited versus adequate English proficiency (4/13, 30% vs 5/7, 71%), limited versus adequate health literacy (7/16, 44% vs 3/4, 75%), and limited versus adequate digital literacy (4/10, 40% vs 6/10, 60%); none of these differences were statistically significant. When examining the proportion of patients who gave extensive feedback for novel messaging content, those with LHL were less engaged than those with adequate health literacy (8/16, 50% vs 4/4, 100%); there were no statistical differences by any subgroup.

**Conclusions:**

Despite widespread mobile phone use, digital literacy barriers are common among vulnerable populations. Engagement in the card sorting activity varied among participants and appeared to be lower among those with LHL, LEP, and LDL. Researchers employing traditional UCD methods should routinely measure these communication domains among their end-user samples. Future work is needed to replicate our findings in larger samples, but augmentation of card sorting with direct observation and audiovisual cues may be more productive in eliciting feedback for those with communication barriers.

## Introduction

### Background

Despite the potential promise of health-related mobile apps in improving the health of individuals with chronic diseases [1-4], few studies evaluate their usability and feasibility, and even fewer do so among vulnerable populations [5-7]. Existing data suggest that populations with limited health literacy (LHL), limited digital literacy (LDL), and limited English proficiency (LEP) are less likely to use health information technology (HIT), including mobile apps [6,8-11]. Given that smartphone ownership rates are similar in low income, minority populations compared with the general population of the United States, there are likely other barriers contributing to this decreased use of health apps [12]. To better understand these barriers and to reduce the disparities in the use of HIT for the management of chronic diseases, it is critical to include the vulnerable populations in the process of app development [13,14].

Even more specifically, there is a need to advance the field in terms of appropriate design science methods for working with vulnerable populations, including racial or ethnic minorities and those with low socioeconomic status and educational attainment [7]. There is similarly a need to understand the use and effectiveness of these methods in patients with mental health disorders that are associated with lower activation and engagement, such as depression [15]. Standard user-centered design (UCD) methods, such as prototyping and card sorting, often use rapid-fire sessions with end users to generate and prioritize a large amount of potential content for a digital health program or intervention [16-18]. Although the goal of these methods is to understand the experiences, beliefs, and preferences of end users, they could represent a cognitively demanding approach for the participants—especially in terms of hypothetical discussions about future health behaviors and sifting through large volumes of potential content.

### Objectives

We sought to understand which design methods worked well within a larger study employing UCD methods to develop a text messaging app aimed at increasing physical activity among patients with comorbid diabetes and depression. In this paper, we evaluate data from 2 sets of semistructured interviews conducted during the early phases of the app development and describe the patterns of mobile phone usage and variations in engagement with design methods by health literacy, English proficiency, and digital literacy of patients recruited from a public health care system.

## Methods

### Research Setting and Sampling Procedure

Data for this study comprise 20 transcripts of semistructured interviews collected during 2016 to 2018 over 2 phases. All patients were recruited from primary care clinics from the public health care system for the city and county of San Francisco. A total of 10 transcripts are from patients who had completed a previous trial evaluating automated text messages as an adjunct to cognitive behavioral therapy for depression (MoodText trial, NCT01083628) [19], and 10 are from a separate group of patients participating in early design sessions to develop a text messaging app to increase physical activity in patients with diabetes and depression (Diabetes and Depression Text Messaging Intervention [DIAMANTE] trial, NCT03490253). Inclusion criteria were as follows: age ≥18 years, English- or Spanish-speaking, ownership of any type of mobile phone, and a diagnosis of depression. Exclusion criteria were active suicidal ideation with a plan and active severe psychosis.

### Data Collection Procedure

Short questionnaires were administered to the participants during recruitment to assess sociodemographic factors (including age, gender, race and ethnicity, education level, income, employment status, and English proficiency), health status, and health literacy. Semistructured, in-depth interviews were conducted with all 20 participants in either English or Spanish. All interviews were 1.5 hours in duration, and the interviews in Spanish were conducted by study staff who were native speakers. Interview guides for all participants included questions about mobile phone usage, physical activity, and feedback on sample text messages and text messaging interventions (interview guides and card sorting instructions are available by request). Participants also completed a closed card sorting activity. Card sorting is a method used to explore how people group concepts, and this has previously been used in the development of text messaging interventions [20]. Participants were given a set of note cards with sample text messages written at a sixth-grade reading level in either English or Spanish, depending on their preferred language, and were asked to sort the cards into 3 piles depending on whether they liked, disliked, or felt neutral about the messages on the cards. They were then asked to explain why they liked or disliked these messages. If they did not provide a reason for liking or disliking the messages, they were probed once more by the interviewer. If the participants did not understand the card sorting activity or had difficulty reading the cards, card sorting was modified such that the interviewer read cards aloud and elicited feedback on each sample text message one at a time. On completion of the card sorting activity, participants were asked if they had any additional ideas for potential text message content or structure of the text messaging intervention.

### Qualitative Analysis

The qualitative analysis was done with open coding of all transcripts using inductive and deductive coding techniques (Dedoose version 8.1.8). One author (SN) created the initial codebook based on the interview questions. A second author (PA) read a subset of transcripts and coded using the original codebook. SN, PA, and CL met frequently to review the codebook, discuss the emerging themes, and resolve disagreements. For this study, we specifically coded for (1) patterns of mobile phone usage and (2) engagement in the design methods. This involved categorizing both the thematic ideas that emerged from the interviews, as well as the extensiveness of participation in (1) the card sorting activity to provide feedback on the existing text messaging content and (2) the semistructured interview to provide novel suggestions for new text messaging content. *Feedback on existing content* was further categorized as *extensive* if it included not only *what* the participants liked or disliked but also *why* they liked it and *how* they felt it would help them. If the feedback on the existing content consisted only of *what* the participants liked or disliked, it was determined to be *minimal*. *Novel suggestions* were *extensive* if they provided detailed content for possible text messages and also offered new types of messages. If novel suggestions were repetitions of text messages already developed by the study and shared with the participant, or if they were unrelated to the purpose or content of the study, they were determined to be *minimal or misaligned*.

### Exploratory Analyses

For our secondary analyses, we were interested in the differences in key themes by participant demographics, specifically, health literacy, English proficiency, and digital literacy. Health literacy was measured using the single-item assessment by Chew et al, “How confident are you filling out medical forms by yourself?” As previously validated in a population similar to that of this study, adequate health literacy was defined by answering “extremely,” whereas LHL was defined by answers “not at all,” “a little bit,” “somewhat,” or “quite a bit” [21-23]. English proficiency was also measured using a validated single-item assessment, collapsing Spanish-speaking participants with those that reported “moderate,” “little,” and “very little ability” with speaking English [24]. Owing to the lack of a validated survey measure of digital literacy that is widely used for mobile phone usage, we created a definition of digital literacy based on the interview responses about the current use of mobile phones. We categorized participants with very limited use or engagement and/or self-reported difficulty in using their mobile phones as having LDL. We based our definition in Kayser et al’s concept of eHealth literacy [25], incorporating both capabilities and experience/engagement in using technologies. We did not include access to technology in our definition as all participants owned a mobile phone. We then quantitatively compared the prevalence of LDL by participants’ health literacy and English proficiency using Fisher exact tests to assess for differences by these 2 independent variables.

Finally, in an exploratory way, we calculated the sum of the number of unique feedback statements or novel suggestions per participant given during the card sorting task. We calculated the means and ranges of the total number of statements given and then preliminary Wilcoxon rank sum tests were conducted to evaluate the differences in these frequencies by health literacy, English proficiency, and digital literacy. We also calculated the number of participants who provided extensive versus minimal feedback statements and novel suggestions and used Fisher exact tests to evaluate the differences in these proportions by health literacy, English proficiency, and digital literacy. Analyses were conducted using Stata/SE 15.0.

## Results

### Participant Characteristics

The average age of the participants was 59 (SD 8) years; 65% (13/20) were female, 90% (18/20) were nonwhite, 65% (13/20) had LEP (50% (10/20) Spanish-speaking), 45% (9/20) had a high school education or less, and 80% (16/20) had LHL (Table 1). None of the participants were employed full time; 45% (9/20) were disabled, and 20% (4/20) were unemployed. A total of 70% (14/20) of the participants had type 2 diabetes mellitus, and 65% (13/20) self-rated their health status as fair or poor. The proportion of participants with LEP (80% vs 50%) and with diabetes (90% vs 50%) was higher in the DIAMANTE trial than the proportion of participants in the MoodText trial; otherwise there were no differences in the demographic or health data by study.

**Table 1:** Participant characteristics (N=20).

Characteristics		Values
Age (years), mean (SD)		59.0 (7.7)
**Gender, n (%)**		
	Men	7 (35)
	Women	13 (65)
**Income (USD), n (%)**		
	<$20,000	11 (55)
	>$20,000	2 (10)
	Other/refused	7 (35)
**Employment status, n (%)**		
	Part time	4 (20)
	Unemployed	4 (20)
	Disabled	9 (45)
	Retired	3 (15)
**Race or ethnicity, n (%)**		
	White	2 (10)
	Hispanic or Latino	11 (55)
	Black or African American	4 (20)
	Asian or Pacific Islander	2 (10)
	Other	1 (5)
Limited English proficiency, n (%)		13 (65)
Limited health literacy, n (%)		16 (80)
**Education, n (%)**		
	None or primary school	7 (35)
	High school graduate or GED^a^	2 (10)
	Some college or technical school	9 (45)
	College graduate or graduate degree	2 (10)
Diabetes, n (%)		14 (70)
Depression, n (%)		20 (100)
**Health status, n (%)**		
	Fair or poor	13 (65)
	Good	7 (35)

^a^GED: General Education Development.

### Mobile Phone Use and Digital Literacy

We uncovered major categories of mobile phone usage related to texting networks, mobile phone carriage, and difficulty with the basic features of mobile phones (ie, text messaging). Overall, nearly all participants (18/20) had a smartphone and used text messaging regularly with a network of family and friends (15/20). A total of 5 participants described having limited texting networks, texting only family members or research studies. Most participants (17/20) reported carrying their mobile phones with them whenever leaving home.

Despite the overall high rate of mobile phone usage, many participants were defined as having LDL. Participants were categorized as having LDL if they had limited texting networks, frequently did not carry their mobile phones or turned them off for long periods of time (limited mobile phone carriage), or had difficulty using their phones or sending text messages owing specifically to unfamiliarity with or difficulty typing or using the microphone feature. Overall, 10 participants were categorized as having LDL; others were categorized as having adequate digital literacy. Those who described difficulty using their phones attributed this primarily to being new smartphone owners and still learning how to use their phones. Several also cited their phones being turned off or forgetting to check their phones, low literacy, and having to use the microphone feature on their smartphone as hindrances to text messaging:

Now that I just got this phone, and before I was able to send text messages but now I’ve forgotten. [...] I didn’t want a high-tech phone […] but my kids gifted me a phone and said, “you have to learn, mom.”

translated from Spanish

I didn’t check my phone or my phone was off for the day. Sometimes I’ll turn my phone off and I forget to turn it back on.

Yes, because I don’t use the keys and sometimes when you speak into the microphone, it doesn’t write the words that you want to say correctly. My daughter calls me out on that. So, if I’m going to send a message that’s not going to be correct like it should be, I’d feel bad.

translated from Spanish

Of the 10 participants with LDL, 7 had LEP and 10 had LHL. There was a similarly high proportion of participants with LEP and/or LHL within each of the mobile phone usage domains we used to define the LDL (Table 2). Specifically, the majority of participants with limited texting network, limited mobile phone carriage, and difficulty using their phone or texting had LEP and/or LHL. Fisher exact test results were not statistically significant.

The other most commonly mentioned barriers to mobile phone usage that were not related to digital literacy included being too busy and forgetting to respond, having a physical impairment (eg, arthritis) that made typing difficult, and not being in the mood to respond to text messages:

Um, I got real busy, I didn’t hear - I didn’t check my phone or my phone was off for the day. Sometimes I’ll turn my phone off and I forget to turn it back on and, um, then it’s late and then I’m sleepy so I won’t answer.

It has such small letters and I have a problem with my hands because I have Raynaud’s syndrome, where your hands fall asleep.

translated from Spanish

Sometimes, well, I don’t pay attention and sometimes I do. Not every day. Sometimes I’m in a bad mood [laughs], sorry for saying it.

translated from Spanish

**Table 2:** Mobile phone digital literacy, as well as domains used to measure digital literacy by English proficiency and health literacy.

Mobile phone digital literacy		Total number of patients, N (%)	LEP^a^, n (%)	LHL^b^, n (%)	Both LEP and LHL, n (%)
**Subdomains of mobile phone digital literacy**		
	Limited texting network	5 (25)	4 (80)	4 (80)	4 (80)
	Limited mobile phone carriage	3 (15)	3 (100)	3 (100)	3 (100)
	Difficulty using phone and/or texting	10 (50)	7 (70)	10 (100)	7 (70)
Overall limited digital literacy		10 (50)	7 (70)	10 (100)	7 (70)

^a^LEP: limited English proficiency.

^b^LHL: limited health literacy.

### Engagement in Card Sorting Activity

Engagement was examined in 2 ways. First, we determined the frequency of feedback given about the existing content and the frequency of novel suggestions provided. We also used our qualitative coding to identify the extensiveness of both feedback on the existing content and novel suggestions.

#### Feedback on Existing Text Message Content

Feedback on text message content was elicited by a card sorting activity—half of the participants required a modification of the activity as they either did not understand it or had difficulty reading the cards in the allotted time.

The most common positive feedback was for the text messaging content to encourage participants to reflect on their behaviors or thoughts, give concrete ideas or advice, or be highly positive and motivating. The most common negative feedback was for the content that was viewed as repetitive. Several exemplar quotes demonstrated these positive and negative feedback themes:

But most of the times when I received the messages, they came at a good time and it just helped me to evaluate, um, you know, my thinking and my feelings, which if it hadn’t came, then I wouldn’t have been thinking about, you know, different things

theme: encourage reflection

These are all concrete suggestions of things that you can try because they will probably improve your mood just because you try. […] So I think it’s just little suggestions or ways that you could help make yourself feel better. Less negative. Less down.

theme: concrete advice

I also liked the way they were positive.

translated from Spanish; theme: positive messages

They’re a little repetitive.

translated from Spanish; theme: repetitive messages

For each participant, the number of different reasons they gave for liking or disliking sample text messages was summed, and this defined the frequency of feedback.

We also evaluated the extensiveness of feedback provided by each participant. Half of the participants provided extensive feedback, as evidenced by these comments:

[They] just make you stop for a minute. I think that’s maybe the key to why it works so well because you know the message comes in and you’re like well why am I doing this? I’m not being very positive today and I could do something better. I could think of another way to handle this. Maybe I should try that.

Well, they spark the idea of getting started. They spark the idea of why - I mean, they have started to continue in giving me I guess motivational nudges here and there.

It says “every time you think you can’t or you won’t be able to [do something], remember the times you were able to.” Yes, there are times that these messages help me, this type of message helps me because often I say, “I can’t,” but occasionally I’ve read these messages and I say, “yes I can. “I’ll try it no matter what,” “I’ll try it,” and then I’ve done it and I have examples.

translated from Spanish

On the other hand, participants who gave feedback that was minimal, provided little, if any, varied feedback, and did not explain why they liked or disliked the text messages even after being probed by the interviewer:

Yeah. Not interested. Okay.

I liked all of them.

translated from Spanish

For quantitative analyses (Table 3), we calculated the frequency of feedback by coding each instance of feedback on the sample text messages. We found that the frequency of feedback was lower in participants with LEP, LHL, and LDL than in those with English proficiency, adequate health literacy, or adequate digital literacy, although none of these differences were statistically significant. It appears that the participants who were proficient in English provided more extensive feedback compared with those with LEP (5/7, 71% vs 4/13, 30%), as did those with adequate versus LHL (3/4, 75% vs 7/16, 44%) and those with adequate versus LDL (6/10, 60% vs 4/10, 40%). These differences approached but did not achieve statistical significance.

**Table 3:** Frequency and extensiveness of the feedback on existing text messaging content by English proficiency, health literacy, and digital literacy.

Patient characteristic		Frequency indicated by median number of unique feedback	Extensiveness indicated by number who provided extensive feedback, n (%)
**English proficiency**			
	Adequate (n=7)	5.5	5 (71)
	Limited (n=13)	4	4 (30)
**Health literacy**			
	Adequate (n=4)	5.5	3 (75)
	Limited (n=16)	4	7 (44)
**Digital literacy**			
	Adequate (n=10)	5	6 (60)
	Limited (n=10)	4	4 (40)

#### Novel Suggestions

Participants were asked whether they had other ideas for text messages that were not presented during the card sorting activity, as well as whether they had any other general feedback on the text messages or the text messaging interventions. The majority of suggestions were for text message content:

Um, or, um, “I know you’re feeling down, so let’s go for a walk and maybe that will pick you up a little bit. I’ve tried that before and it happens, so let’s do it.”

Hmm. “Rise and shine!” Uh, let’s see, okay. “Rise and shine, get off your behind!”

A fewer number of participants provided feedback on broader, structural content of the text messaging interventions:

It could be a little cartoon running, an example of someone walking or running.

translated from Spanish

There are thousands of illnesses in the world. So, first you need to know which illnesses someone has, and what difficulties they may have in doing physical activity. There are many types of physical activity. Because after my surgery my exercise was to stretch, to lift my arms with a pound of rice. It’s moving the body. From here down I couldn’t move because of my knee. Stretching, nothing more. Of course. This way, you have to see what the patient has to motivate them.

translated from Spanish

We evaluated the extensiveness of novel suggestions as well. Extensive novel suggestions were defined as providing detailed content for possible text messages and also offering new types of messages:

And I would send them [a message] saying that life is very beautiful. Life is a thing of beauty and one should be happy and put aside the negative to give life to the positive.

translated from Spanish

That the gym is not the only place where one can exercise, because you have this mentality of “I’ll sign up but it’s very expensive.” If there are other options for exercise, that’s excellent.

translated from Spanish

Suggestions were categorized as *minimal or misaligned*, if they were vague or identical to the messages presented during card sorting or were unrelated to the physical activity content focus of the text messaging intervention (eg, text messages with the results of laboratory tests):

Probably reminding uh reminding me for doing me or exercise, walking, whatever.

I think that if you're going to send me one of these messages, it’s to warn me about something that happened or is about to happen. I mean to say […] let me give an example. The doctor saw […] my results and wanted to tell me how they went.

translated from Spanish

We coded each separate answer by participants as *novel suggestions*, and the frequency of novel suggestions for each participant was defined as the sum of the coded excerpts. The median number of novel suggestions was 2 per participant (range 0-9; Table 4), and there were no significant differences by English proficiency, health literacy, or digital literacy. All participants with adequate health literacy provided extensive novel suggestions, compared with only half of those with LHL (Table 4). There were no differences in the extensiveness of novel suggestions by English proficiency or digital literacy.

**Table 4:** Frequency and extensiveness of novel suggestions by English proficiency, health literacy, and digital literacy.

Patient characteristic		Frequency indicated by median number of suggestions	Extensiveness indicated by number that provided extensive suggestions, n (%)
**English proficiency**			
	Adequate (n=7)	2.5	4 (57)
	Limited (n=13)	2	6 (56)
**Health literacy**			
	Adequate (n=4)	2	4 (100)
	Limited (n=16)	2	8 (50)
**Digital literacy**			
	Adequate (n=10)	2	5 (50)
	Limited (n=10)	2	5 (50)

## Discussion

### Principal Findings

Our study found that the participants representing vulnerable populations engaged differently in the UCD process that we employed and described difficulty with mobile phone usage and text messaging. Although nearly all participants had a smartphone that they carried with them throughout the day and a large majority had wide texting networks, over three-quarters still described difficulty with text messaging. This discrepancy suggests that smartphone ownership, even with daily personal use, does not accurately predict comfort or ability in using the basic features of phones such as text messaging. This finding is supported by emerging data demonstrating limited use (eg, voice communication only) of mobile phones by vulnerable populations [26], as well as by efforts in mobile interaction design to focus on low literacy or illiterate populations [27].

We found variations in engagement in our design process by health literacy, English proficiency, and digital literacy. Digital literacy, defined broadly by the US Department of Education as “digital problem solving” and measured by assessments of basic computer competence, affects 16% of the US population; those who have LDL tend to be older, nonwhite, foreign born, and less educated than those who are digitally literate [28]. This definition, however, does not incorporate the use of other ubiquitous technologies, including smartphones, and has not been evaluated in the context of health and health care. Therefore, we used a de novo classification from the participants’ responses to calculate our own definition of mobile phone digital literacy, informed by previous conceptual models in this space. Most individuals we identified as having LDL also had LHL and LEP, suggesting some overlap in these constructs. User characteristics such as age, education, and employment are considered in frameworks for developing information technology systems, and it is likely that health literacy and English proficiency are similarly important user characteristics in health-related digital literacy [25,29]. In fact, health literacy has been included as a component of digital literacy in some conceptual models [30].

Despite following best practice guidelines for card sorting, such as limiting the number of cards and categories and providing uniform, clear directions on how to conduct the task [17,31,32], this method had to be modified in half of the interviews and was therefore not fully effective in this study population. Eliciting novel suggestions was also difficult, with most participants only providing 1 or 2 suggestions. The latter is consistent with studies of engagement in qualitative methods that suggest that end users are often unable to provide anticipatory feedback [16]. However, we expected more consistency in the feedback on existing materials, as end users are usually more engaged in this type of feedback. This may be because in obtaining feedback via a card sorting method, we created the additional challenge of having to provide feedback on hypothetical messages within a set time limit, which has been shown to be a more challenging task [16]. It is also possible that the participant’s response bias owing to the interviewer’s demand characteristics contributed to the differences in feedback by health and digital literacy and English proficiency, though we tried to minimize this by conducting interviews in Spanish with native Spanish-speaking interviewers [33]. Future work is needed to replicate our findings in larger samples; however, our findings suggest that augmenting card sorting with different methods in UCD, such as task analysis and usability testing with direct observation, may be more effective in obtaining feedback from vulnerable populations [18,25,29]. Furthermore, observation in environments in which end users are both comfortable and will be using the digital health intervention may be more productive in eliciting feedback and matching the intervention with the end users’ needs and preferences.

In the development of digital health interventions to improve outcomes in patients with diabetes, others have also employed user-centered or participatory design and have obtained and successfully incorporated feedback in their development processes [34,35]. Although these studies have enrolled diverse populations, they do not describe whether the feedback they received differed within subgroups of their populations. Our exploratory analyses showed differences in engagement in specific subgroups of patients, suggesting that there may be other factors contributing to these differences. LHL, specifically, has been associated with lower patient activation and engagement in clinical settings, and this may be relevant in research settings as well [36-38]. Our findings underscore the importance of measuring health literacy, English proficiency, and digital literacy and considering their effects in UCD. This is critical as simply recruiting these vulnerable populations to user-centered or participatory design research rather than engaging them in feedback processes in a meaningful way carries the risk of exacerbating existing disparities in the use of HIT [8].

### Limitations

There are several limitations to this study. In defining engagement, we assessed the extensiveness of feedback statements and novel suggestions; however, the provision of minimal feedback may have been owing to disinterest in the content being discussed rather than purely a lack of engagement. Given our small sample sizes that are typical in design research [31,32], many comparisons did not reach statistical significance; nevertheless, our quantitative data did allow us to highlight these differences. In addition, we focused specifically on mobile phone–related digital literacy rather than a more comprehensive digital literacy assessment, given the focus of our future text messaging intervention. Furthermore, 10 of the participants had already participated in a trial evaluating a text messaging app and therefore had more familiarity with this type of intervention. Nevertheless, neither successful completion of the card sorting task nor the engagement differed by study. Finally, although we chose to look specifically at health literacy, English proficiency, and digital literacy as the predictors of engagement, there are almost certainly other factors contributing to the differences in engagement noted in these populations, including education, race, culture, patient activation, and the nature and homogeneous content of the interviews [37,39]. Notably, all the study participants had depression, which has been associated with lower patient activation [40]; however, despite this, we were still able to detect differences in engagement within subgroups of the population.

### Conclusions

Engagement in our design process varied by health literacy, English proficiency, and digital literacy. The participants of our study represent a diverse population—in race, employment, education, literacy and language, and general health status—that is rarely captured in usability studies for HIT. We believe this to be a major strength of this paper, both in describing the variations in patterns of mobile phone usage and in evaluating the engagement in UCD methods. Our findings highlight the need for a better understanding of how to consider, define, and incorporate digital literacy when developing HIT, as well as continued efforts in better engaging vulnerable populations in research. Our future work will report on the process for incorporating format and content feedback into our final intervention.

## Abbreviations

DIAMANTE: Diabetes and Depression Text Messaging Intervention
HIT: health information technology
LDL: limited digital literacy
LEP: limited English proficiency
LHL: limited health literacy
UCD: user-centered design
